# A comparison of predictors for mortality and bacteraemia in patients suspected of infection

**DOI:** 10.1186/s12879-021-06547-0

**Published:** 2021-08-23

**Authors:** Steen Andreassen, Jens Kjølseth Møller, Noa Eliakim-Raz, Gorm Lisby, Logan Ward

**Affiliations:** 1 Treat Systems ApS, Ålborg, Denmark; 2 Department of Health Science and Technology, Aalborg University, Ålborg, Denmark; 3 Department of Clinical Microbiology, University Hospital of Southern Denmark, Lillebælt Hospital, Vejle, Denmark; 4 Department of Medicine E, Beilinson Hospital, Rabin Medical Centre, Petah Tiqva, Israel; 5 Sackler Faculty of Medicine, Tel-Aviv University, Tel-Aviv, Israel; 6 Department of Clinical Microbiology, University Hospital of Copenhagen, Amager og Hvidovre Hospital, Hvidovre, Denmark

**Keywords:** Decision support, Risk-based stratification, Sepsis, Bacteraemia, Predictive models

## Abstract

**Background:**

Stratification by clinical scores of patients suspected of infection can be used to support decisions on treatment and diagnostic workup. Seven clinical scores, SepsisFinder (SF), National Early Warning Score (NEWS), Sequential Orgen Failure Assessment (SOFA), Mortality in Emergency Department Sepsis (MEDS), quick SOFA (qSOFA), Shapiro Decision Rule (SDR) and Systemic Inflammatory Response Syndrome (SIRS), were evaluated for their ability to predict 30-day mortality and bacteraemia and for their ability to identify a low risk group, where blood culture may not be cost-effective and a high risk group where direct-from-blood PCR (dfbPCR) may be cost effective.

**Methods:**

Retrospective data from two Danish and an Israeli hospital with a total of 1816 patients were used to calculate the seven scores.

**Results:**

SF had higher Area Under the Receiver Operating curve than the clinical scores for prediction of mortality and bacteraemia, significantly so for MEDS, qSOFA and SIRS. For mortality predictions SF also had significantly higher area under the curve than SDR. In a low risk group identified by SF, consisting of 33% of the patients only 1.7% had bacteraemia and mortality was 4.2%, giving a cost of € 1976 for one positive result by blood culture. This was higher than the cost of € 502 of one positive dfbPCR from a high risk group consisting of 10% of the patients, where 25.3% had bacteraemia and mortality was 24.2%.

**Conclusion:**

This may motivate a health economic study of whether resources spent on low risk blood cultures might be better spent on high risk dfbPCR.

## Introduction

In the Emergency Department (ED) about 40% of patients admitted to hospital [1] may be suspected of infection. For these patients the decisions of immediate interest for the diagnostic work-up are whether a blood culture should be drawn and how many resources the microbiology lab should spend on providing a rapid answer. Rapid microbiology diagnostics decrease time to identification of pathogens and potentially enable earlier initiation of targeted antimicrobial therapy improving antimicrobial stewardship programs [2]. For example, when a blood culture becomes positive, identification of pathogens by MALDI-TOF MS directly from positive blood cultures are now routine in many labs. Sub-species typing, and detection of drug resistance determinants besides microbial identification from isolated colonies are also being explored for MALDI-TOF MS [3]. Another decision is whether blood cultures should be supplemented by a more expensive, but much faster, method based on direct-from-blood PCR (dfbPCR) [4]. Mangioni et al. proposed the use of multiparemeter scores to triage patients for rapid diagnostic procedures [5], where scores which can predict the likelihood of a useful answer (probability of bacteraemia) and the need for rapid result (high probability of mortality) may be useful. Ordering blood cultures without considering the pretest probability may be both wasteful and harmful [6].

Clinical scores which can predict the probability of bacteraemia such as those described in two reviews [7, 8] can help make these decisions, including whether dfbPCR should supplement blood culture in some patients [9].

Several clinically validated scores have been used to predict mortality in ED patients, such as the National Early Warning Score (NEWS) [10] and the Mortality in Emergency Department Sepsis (MEDS) [11]. The Systemic Inflammatory Response Syndrom (SIRS) [12] was established to define operational criteria for a sepsis diagnosis. SIRS has been replaced by the Sequential Organ Failure Assessment (SOFA) score or by the quick-(q-) SOFA score in the Sepsis-3 consensus definition of sepsis [13]. The Shapiro Decision Rule (SDR) predicts bacteraemia for ED patients [14] as does SepsisFinder (SF) [15].

The primary objective of this study is to retrospectively compare predictions of 30-day mortality and bacteraemia from all of these scores: SF, NEWS, SOFA, MEDS, qSOFA, SDR and SIRS. All scores will be assessed based on their Area Under the Receiver Operating Characteristic (AUROC) curves.

The review by Coburn et al. [7] focuses on overuse of blood culture in low-risk patients, which may be due to an overestimation of the probability of bacteraemia by physicians [16]. They conclude that both SIRS and SDR perform well in identifying a low risk group which may not need blood culture. Pawlowicz et al. [17] found a 33.5% reduction in the number of ordered blood cultures after implementation of SDR. Another evaluation of SDR found that it was able to select a group of 45% of all patients that had a bacteraemia rate of only 0.9% [18].

In line with these studies, a secondary objective of this study will be to compare how well each of the scores can identify a low-risk group, consisting of about one third of the patients, where blood culture may be of limited value. In addition we will identify a high-risk group, consisting of 10% of the patients, where dfbPCR may be justifiable, despite its relatively high cost.

## Methods

### Patient data

The three test datasets will be referred to as HvH, SLB and TREAT04.

#### HvH

263 patients with suspected sepsis at Hvidovre Hospital, Hvidovre, Denmark; November 2011 to April 2012 [19].

#### SLB

199 patients with suspected sepsis at Lillebælt Hospital, Vejle, Denmark; July to August 2012 [20].

#### TREAT04

1354 patients admitted to a department of medicine with suspected community acquired infections at Rabin Medical Center, Petach Tikva, Israel. Data were collected in an interventional study of TREAT from May to November 2004 [21].

### SF predictions

SF [15] is a CPN (Causal Probabilistic Net or Bayesion Net) model of part of the inflammatory response. It uses age, temperature, heart rate, calculated mean arterial pressure, mental status, neutrophil fraction, platelets, CRP, lactate, creatinine and albumin as input variables. The outputs from SF are 30-day mortality and the probability of bacteraemia. It is an inherent part of the CPN technology that SF tolerates missing values well. Input data for calculation of the SF prediction of bacteraemia will therefore be considered “complete” if any three out of the 11 possible input variables are available. Age is not used for the prediction of bacteraemia. The prediction of mortality uses the same input variables as the bacteraemia prediction, plus age as an independent factor [22]. The SF CPN was implemented in Hugin (version 8.7, Hugin Expert A/S), commercially available software for constructing and using CPNs. SF was trained on one dataset and tested on three independent datasets [15]. These three datasets (HVH, SLB and TREAT04) will also be used in this study in the comparison of performance between SF and the other clinical scores.

### Clinical scores

Scores commonly used to aid diagnosis and/or prognosis in patients with suspected sepsis were included: NEWS, SOFA, MEDS, qSOFA, SDR and SIRS. The data items required for calculation of the clinical scores are given in Table 1. To best accommodate the data requirements of the different scores, data were mapped when required and possible.

**Table 1:** Data items used to calculate the clinical scores

Variables	SF	Variables used to calculate scores					
		NEWS	SOFA	MEDS	qSOFA	SDR	SIRS
Vital signs							
Systolic BP or MAP	x	x	x		x	x	
Heart rate	x	x					x
Temperature	x	x				x	x
Chills	x					x	
Mental status	x	x		x	x		
GCS							
Arterial O_2_ saturation (SaO_2_)		x					
Arterial O_2_ pressure (PaO_2_)			x				
Respiratory rate		x			x		x
Respiratory distress				x			
Laboratory							
WBC						x	x
Platelets	x		x	x		x	
Creatinine	x		x			x	
Neutrophil fraction	x						
Immature neutrophils				x		x	
Bilirubin			x				
Albumin	x						
CRP	x						
Lactate	x						
Other/comorbidities							
Age	x			x		x	
Nursing home residence				x			
Lower respiratory infection				x			
Septic shock				x			
Terminal illness				x			
Suspected endocarditis						x	
Indwelling vascular catheter						x	
Vomiting						x	
Overall score availability (%)	99.8	33.9	23.5	69.1	50.8	61.8	58.5

### Calculated/mapped variables

Glasgow coma scale (GCS) was not available in the datasets. However, mental status was recorded as normal, confused or comatose. Normal was mapped to alert (GCS = 15), both confused and comatose were mapped to not alert (GCS < 15). PaO_2_ was less widely recorded than SaO_2_, so to give additional availability for SOFA which requires PaO_2_, a mapping was made from SaO_2_. Respiratory distress as used in MEDS was calculated if at least one of respiratory rate and SaO_2_ were present in the dataset.

### Adjustments to the scores

Other than the use of the mapped variables described, no adjustments to the scoring methods were made for NEWS, qSOFA and SIRS. Terminal illness and immature neutrophils were not recorded in any of the datasets. Therefore MEDS was calculated assuming these variables did not contribute to the scores in patients where they were missing. Adjustments were also made to the SOFA score: we did not require evidence of mechanical ventilation for the respiratory component, the maximum score of the cardio component was 1 due to lack of information on vasopressors, the maximum CNS score was 1, using alert/not alert as the GCS score was not available and the renal component was calculated without use of urine output.

### Completeness

For NEWS, MEDS, SOFA, qSOFA and SIRS the scores were only calculated if the data for the patients were complete. Data were considered complete where all of the variables used in the adjusted scores were present. SF was used with incomplete data, provided at least three of the 11 possible variables for SF were available.

### Microbiology

Bacteraemia was defined as positive blood cultures with one or more clinically significant pathogen. *Bacillus* spp. (except *B. anthracis*), coagulase-negative staphylococci (CoNS), *Corynebacterium* spp. and *Micrococcus* spp. were considered contaminants in the absence of other clinical evidence.

### Outcomes and statistical analysis

The primary outcomes were bacteraemia and all-cause 30-day mortality. Predictive performance was assessed by AUROC. AUROCs were compared using the method of De Long [23] as implemented in the pROC package of R (R version 3.5). To simulate possible clinical scenarios, two cut-offs were determined for each score that would result in a low-risk group of approximately one third of patients, and a high risk group of approximately 10% of patients. Outcomes in each risk group were assumed to be binomially distributed. Confidence intervals for binomial proportions were calculated under the assumption of normality. Analyses were performed in R (version 3.5) and Python (version 3.7), visualizations were constructed using Matplotlib [24].

## Results

### Descriptive statistics

Table 2 presents the demographics for each of the included datasets as well as for the data material as a whole.

**Table 2:** Demographic description of datasets

	SLB	HvH	TREAT04	Overall
Patients, n	199	263	1354	1816
Female, n/N (%)	90/199 (45.2)	142/263 (54.0)	658/1328 (49.5)	890/1790 (49.7)
Age, median [IQR]	67 [49–79]	75 [58–84]	72 [56–81]	72 [55–81]
Place of acquisition, n (%)				
Community	184 (93.9)	250 (95.1)	1354 (100)	1788 (98.4)
Nursing home	12 (6.1)	11 (4.2)	0 (0)	23 (1.3)
Hospital	0 (0)	2 (0.8)	0 (0)	2 (0.1)
Final diagnosis, n (%)				
Urinary tract infection	34 (17.1)	38 (14.6)	249 (18.4)	321 (17.7)
Pneumonia/LRT	33 (16.6)	158 (60.5)	295 (21.8)	486 (26.8)
Skin/soft tissue infection	14 (7.0)	10 (3.8)	149 (11.0)	173 (9.5)
Abdominal infection	30 (15.1)	0 (0)	84 (6.2)	114 (6.3)
Other infection	31 (15.6)	8 (3.1)	381 (28.1)	420 (23.1)
Non-infectious	57 (28.6)	49 (18.6)	196 (14.5)	302 (16.6)
Outcomes, n (%)				
Positive blood culture				
Bacteraemia	13 (6.5)	18 (6.8)	126 (9.3)	157 (8.7)
Contaminant	15 (7.5)	5 (1.9)	65 (4.8)	85 (4.7)
30-day mortality	13 (6.5)	23 (8.7)	155 (11.4)	191 (10.5)

### Data availability

Table 3 gives the data availability for the three datasets, as well as for the combined dataset, consisting of all three datasets. The table reflects local differences in clinical practice. For example PaO_2_ and SaO_2_ were well recorded for SLB and HvH, but not for TREAT04. In general, vital signs such as blood pressure, heart rate and temperature were well recorded, while mental status and respiratory rate were recorded less often. If we require that all data involved in a clinical score must be recorded, most of the scores could only be calculated for very few patients. MEDS could not be calculated for any patients because none of the datasets contained data on immature neutrophils or on terminal illness.

**Table 3:** Data availability for the data sets (%)

Variables	SLB (n = 199)	HvH (n = 263)	TREAT04 (n = 1354)	Combined (n = 1816)
Vital signs				
Systolic BP or MAP	100.0	98.1	99.9	99.7
Heart rate	100.0	97.0	99.5	99.2
Temperature	96.5	96.2	99.9	99.0
Chills	98.5	81.0	70.1	74.8
Mental status or GCS	88.4	56.3	83.8	80.3
SaO_2_	99.5	95.4	59.7	69.2
PaO_2_ (incl. mapped from SaO_2_)	52.8 (99.5)	24.0 (95.8)	9.5 (60.2)	16.4 (69.6)
Respiratory rate	100.0	53.6	57.8	61.8
Respiratory distress	100.0	97.7	84.1	87.8
Laboratory				
WBC	100.0	74.9	98.8	95.5
Platelets	95.0	74.1	98.4	94.5
Creatinine	99.5	66.9	97.9	93.6
Neutrophil fraction	86.4	73.8	98.4	93.5
Band cells	NR	NR	NR	NR
Bilirubin	87.9	66.9	35.2	45.6
Albumin	99.5	66.5	32.2	44.5
CRP	100.0	67.7	3.2	23.1
Lactate	52.8	21.7	11.9	17.8
Other/comorbidities				
Age	100.0	100.0	100.0	100.0
Nursing home residence	98.5	100.0	100.0	99.8
Lower respiratory infection	100.0	100.0	100.0	100.0
Septic shock	54.3	78.7	94.5	87.6
Terminal illness	NR	NR	NR	NR
Suspected endocarditis	100.0	100.0	100.0	100.0
Indwelling vascular catheter	100.0	100.0	100.0	100.0
Vomiting	75.4	58.6	99.9	91.2

*NR* not recorded in the dataset

### Similarity between datasets

The predictive performance for mortality, measured by the area under the ROC curve (AUROC), was calculated for SF and for the 5 clinical scores for each of the three datasets, as well as for the combined dataset. Tables 4 and 5 show the percentage of complete cases and AUROCS for all datasets and scores for mortality and bacteraemia, respectively.

**Table 4:** Percent complete cases and AUROC for mortality for all datasets and scores

	Mortality								
	SLB (n = 199)		HvH (n = 263)		TREAT04 (n = 1354)		Combined (n = 1816)		
	% compl	AUROC	% compl	AUROC	% compl	AUROC	% compl	AUROC	AUROC(SF)
SF	100.0	0.804	98.9	0.838	100.0	0.761	99.8	0.775	–
NEWS	84.9	0.835	35.4	0.671	26.1	0.704	33.9	0.734	0.786
SOFA	75.9	0.808	24.0	0.707	15.7	0.650	23.5	0.721	0.779
MEDS	46.2	0.867	39.5	0.698	78.8	0.705	69.1	0.710*	0.777
qSOFA	88.4	0.735	36.9	0.728	48.0	0.665	50.8	0.681*	0.790
SDR	61.8	0.473	31.2	0.630	67.7	0.576	61.8	0.587*	0.781
SIRS	96.5	0.559	36.5	0.608	57.2	0.559	58.5	0.566*	0.780

Scores listed in order of declining AUROC for the combined dataset

^*^This AUROC is lower than the AUROC (SF) (p < 0.005)

**Table 5:** Percent complete cases and AUROC for bacteraemia for all datasets and scores

	Bacteraemia								
	SLB (n = 199)		HvH (n = 263)		TREAT04 (n = 1354)		Combined (n = 1816)		
	% compl	AUROC	% compl	AUROC	% compl	AUROC	% compl	AUROC	AUROC(SF)
SF	100.0	0.804	98.9	0.722	100.0	0.737	99.8	0.745	–
NEWS	84.9	0.666	35.4	0.733	26.1	0.712	33.9	0.694	0.738
SOFA	75.9	0.825	24.0	0.467	15.7	0.692	23.5	0.719	0.736
MEDS	46.2	0.603	39.5	0.525	78.8	0.558	69.1	0.558*	0.736
qSOFA	88.4	0.593	36.9	0.665	48.0	0.591	50.8	0.599*	0.761
SDR	61.8	0.798	31.2	0.803	67.7	0.735	61.8	0.743	0.739
SIRS	96.5	0.607	36.5	0.608	57.2	0.643	58.5	0.636*	0.741

^*^This AUROC is lower than the AUROC(SF) (p < 0.001)

To determine the suitability of combining the datasets, the AUROCs for each dataset were compared to the AUROC for the remainder of the combined dataset. Out of these 36 comparisons none were significantly different (p < 0.05) from the SOFA score for the combined dataset. It thus seems that SF and the 6 clinical scores perform similarly for all data sets. This indicates that the comparisons performed in the next section between the performance of SF and the 6 clinical scores can be done, using the combined dataset.

### The performance of SF and the clinical scores

**Fig. 1:**
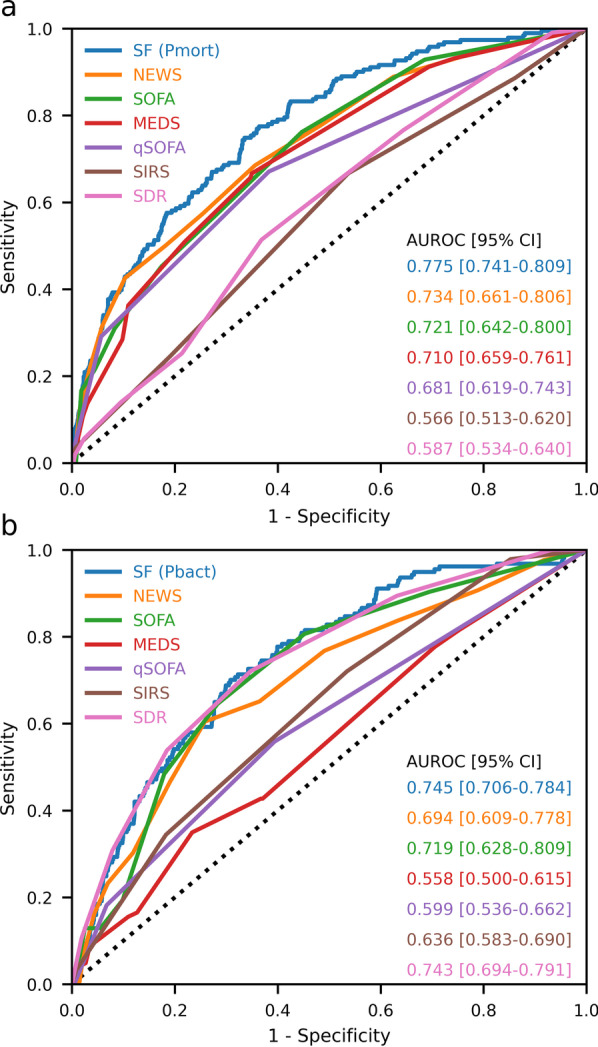
ROC curves for complete cases for each score for the combined dataset. **A** ROC curve for 30-day mortality, **B** ROC curve for bacteraemia

The performance of SF and the 6 clinical scores will be assessed from their ROC curves for mortality and bacteraemia. In Tables 4 and 5 the columns labelled AUROC(SF) contain AUROCs for SF, but only calculated for complete cases for the test considered in the row. Pairwise comparisons of mortality AUROCs for SF with AUROCS for each of the clinical scores showed that the AUROCs for SF were significantly higher that the AUROCs for MEDS, qSOFA, SDR and SIRS (p < 0.005) and was not significantly different from AUROCs for NEWS and SOFA. Figure 1a shows the mortality ROC curves for each of the scores.

The bacteraemia AUROCs for SF were significantly better that the AUROCs for MEDS, qSOFA and SIRS (p < 0.005) and was not significantly different from AUROCs for NEWS, SOFA and SDR. Figure 1b shows the bacteraemia ROC curves for each of the scores.

SF performed better than the scores by having a significantly larger number of complete cases, due to SFs tolerance of missing data. Table 6 offers an alternative way of looking at the clinical scores’ vulnerability to missing data. In Table 6 mortality and bacteraemia AUROCs were calculated for SF, NEWS, SOFA and SDR both for complete cases and for all cases in the combined dataset. In the calculation for all cases, the missing variables were assumed to be non-pathological for the clinical scores. When calculated for all cases, the AUROCs for NEWS and SOFA became significantly smaller than the AUROC for SF, both for mortality and bacteraemia. For SDR this also applied to the mortality AUROC, but not to the bacteraemia AUROC.

**Table 6:** Comparison of AUROC assuming missing = normal for clinical scores

	AUROC [95% CI], n = 1816			
	30-day mortality		Bacteraemia	
	Complete	All	Complete	All
SF	0.775	0.775	0.745	0.745
NEWS	0.734	0.680*	0.694	0.607*
SOFA	0.721	0.671*	0.719	0.614*
SDR	0.587*	0.584*	0.743	0.740

^*^p < 0.001 vs. SF

### Low risk and high-risk groups for bacteraemia

Two examples of clinical decisions where it may be useful to use the scores to stratify patients suspected of infection will be considered. The first scenario concerns the omission of blood culture in low risk patients. The second scenario concerns a potential introduction of dfbPCR in high risk patients.

### Omission of blood cultures in low risk patients

**Fig. 2:**
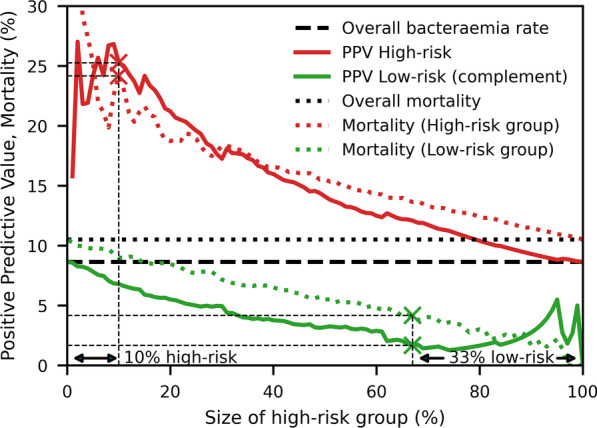
PPV for SF’s bacteraemia prediction and mortality vs. the size of the high risk group

In the low risk scenario, the scores will be used to select a low risk group, consisting of about a third of the patients, those with the lowest predicted probability of bacteraemia. For SF the percentage of patients with bacteraemia in the low risk group can be read to 1.67% from Fig. 2 (green cross on the curve in labelled PPV low-risk). Assuming a cost of € 33 for a blood culture [25] then the cost of obtaining one positive blood culture will be € 33/1.67% = € 1976 in the low risk group. The mortality for the low risk group was 4.2% (green cross on dotted line in Fig. 2. Table 7 shows that compared to the other scores, SF gives the lowest probability of bacteraemia. In this low risk group the were 22 false positive blood culture, corresponding to a contaminant rate of 3.7%.

**Table 7:** Performance of scores in the low risk patients

Score	Low risk (%)	Bact. rate μ ± sd (%)	Mortality μ ± sd (%)	Cost per positive blood culture (€)
SF	33.0	1.67 ± 0.52	4.2 ± 0.8	1976
NEWS < 3	35.2	3.23 ± 1.20	2.8 ± 1.1	1023
SOFA < 1	29.0	2.42 ± 1.38	2.4 ± 1.4	1364
MEDS < 3	28.9	6.30 ± 1.27	2.5 ± 0.8	524
qSOFA < 1	59.3	6.22 ± 1.03	4.8 ± 0.9	531
SDR < 2	34.4	2.86 ± 0.85	6.5 ± 1.3	1158
SIRS < 2	44.9	5.45 ± 1.04	8.0 ± 1.2	605
None^a^	33.0	8.65 ± 1.15	10.5 ± 1.3	382

The cut-off values were chosen to give a low risk group as close a possible to 33% of all patients

^a^Equivalent of randomly selecting 33%

### dfbPCR in high risk patients

In the high risk scenario, the scores will be used to select a high risk group, consisting of about 10% of the patients with the highest predicted probability of bacteraemia. For SF, the percentage of patients with bacteraemia in the high risk group can be read as 25.3% from Fig. 2 (red cross on the curve labelled PPV high-risk). The cost of the two dfbPCR that have been on the market, SeptiFast and Iridica is €127 and €373 [26], respectively. For the cheapest of these, the cost of obtaining one dfbPCR positive bacteraemia case (excluding contaminants) will be €127/25.3% = € 502, assuming that the rate of DNAaemia is the the same as the rate of bacteraemia. The mortality of the patients in the high risk group can be read to 25.3% (red cross) from the graph labelled mortality low risk. Table 8 shows that for all the clinical scores the cost of detecting one bacteraemia case with dfbPCR in the high risk group is smaller than the cost (€ 1976) of detecting one bacteraemia case by blood culture in the low risk group, as defined by SF in Table 7.

**Table 8:** Performance of scores in the high risk patients

Score	High risk (%)	Bact. rate μ ± sd (%)	Mortality μ ± sd (%)	Cost per positive dfbPCR (€)
SF	10.0	25.3 ± 3.2	24.2 ± 3.2	502
NEWS > 8	8.1	20.0 ± 5.7	34.0 ± 6.7	635
SOFA > 4	10.5	13.3 ± 5.1	28.9 ± 6.8	952
MEDS > 7	10.9	11.2 ± 2.6	20.3 ± 3.4	1135
qSOFA > 1	7.7	19.7 ± 4.7	32.4 ± 5.6	644
SIRS > 2	19.7	15.3 ± 2.5	13.4 ± 2.4	829
SDR > 4	10.0	28.6 ± 4.3	13.4 ± 3.2	444
None	10.0	8.7 ± 2.1	10.5 ± 2.3	1469

## Discussion

For the combined dataset SF obtained mortality AUROCs, calculated from cases with complete data, of 0.775 which was higher than for NEWS (0.734) and SOFA (0.721) and significantly higher than for MEDS, qSOFA, SIRS and SDR.

For the combined dataset SF obtained bacteraemia AUROCs of 0.745, higher than for SDR (0.743), SOFA (0.719) and NEWS (0.694) and significantly higher than for MEDS, qSOFA and SIRS.

SF could identify a low risk group, consisting of about one third of the patients. In that group the bacteraemia rate was 1.7% and the average price of obtaining one positive blood culture was quite high, € 1976.

SF could also identify a high risk group, consisting of 10% of the patients. In that group the bacteraemia rate was 25.3%. The cost of obtaining one positive identification of a pathogen by dfbPCR was estimated to € 502, despite the relatively high cost of dfbPCR. Interestingly this cost is substantially lower than the cost of obtaining a positive blood culture in the low risk group.

The study was based on three data sets HVH, SLB and TREAT04. These data sets have the strength that they are diverse. They were collected over almost a decade, in countries with high and low antimicrobial resistance, with a large variation in the amount and type of data collected and with substantial differences in mortality. This demonstrated the robustness of both SF and the clinical scores in the sense that they all showed uniform performance across these differences.

These differences also gave some weaknesses of the study: Although the scores seem to be able to stratify the patients across the differences, it may prove necessary to adjust cut-off values to adapt to the dataset at hand. Another weakness of the datasets was that in many patients only some of the scores could be calculated. This weakened the data, which already suffered the limitation of the small size of the Danish datasets. It does, however, highlight the tolerance of SF to missing data, since SF could be applied for virtually all data in the data sets.

The age of the data is also a weakness, since data on sepsis markers as procalcitonin and CRP were either absent or scarce in the oldest of the datasets, TREAT04. CRP is one of the stronger sepsis markers in the dataset used to train the SF model and although SF performs better than any single data item [15] it is to be expected that more CRP measurements would have improved the performance of SF. This may be even more true of procalcitonin.

In the literature AUROCs were found for SOFA and qSOFA for in-hospital mortality in a large validation dataset: AUC = 0.74 (all) and 0.79 (non-ICU) for SOFA and 0.66 (all) and 0.81 (non-ICU) for qSOFA [27]. Similar results are observed for recent studies outside the ICU with AUROC ranging from 0.77–0.83 for SOFA [28–32] and 0.63–0.77 for qSOFA [29, 30, 33–35]. MEDS is also a predictor of mortality.It had an AUC of 0.82 and 0.76 for its derivation and validation cohorts, respectively [11], although significant variability has been seen in the literature with AUC ranging from 0.67–0.77 in five recent studies [36–41]. NEWS also performs well as a predictor of mortality, with reported AUROC between 0.67–0.78 [30, 32, 35, 42].

Use of standard clinical scores as predictors of bacteraemia is not well reported in the literature. A review identified several validated models, including SDR, although noted that very few scores for bacteraemia were prospectively validated and performed well, and none were in routine clinical use [8]. The other scores included in the analysis have not been evaluated specifically for prediction of bacteraemia outside of isolated studies. In one study, qSOFA showed some potential in a subgroup of elderly patients, however the overall AUROC was 0.64 [43]. The same study reported an overall AUROC of 0.60 for SIRS.

The clinical applications discussed in the paper may deserve a health-economic evaluation. The cost estimates for the low risk group indicate that blood culture from a low risk group may not be cost-effective, in particular because the testing of this group gave rise to 3.7% false positive blood cultures, which is higher than the 1.7% true positive blood cultures. As noted by Bates et al. [6] these are presumably associated with substantially increased cost due to increased length af stay (4.5 days) and increased consumption of antibiotics (39%) and the true costs of contaminants may greatly exceed those of the test itself.

In contrast, dfbPCR from high risk patients may be cost effective in terms of a rapid diagnosis. Realloction of resources currently spent on blood cultures from low risk patients to dfbPCR from high risk patients may be a cost neutral way of improving the quality of microbiological services. However, a prospective randomized clinical outcome study is warranted in order to routinely apply any risk assessment tool for eliminating any currently applied diagnostic intervention in any patient group, including the omittance of blood culture in a patient population scoring low on sepsis risk.

## Conclusions

SF performed better than the clinical scores for prediction of mortality and bacteraemia, significantly so for MEDS, qSOFA and SIRS. For mortality predictions SF was also significantly better than SDR.

In a low risk group consisting of one third of the patients the cost of one positive result from blood culture was € 1976, which was higher than the cost of € 514 of one positive dfbPCR from a high risk group consisting of 10% of the patients. This may motivate a health economic study of whether resources spent on low risk blood cultures might be better spent on high risk dfbPCR.

## Data Availability

The datasets analyzed during the current study are available from the corresponding author on reasonable request with permission from the respective Data Controllers. Statistical code is available on reasonable request. The SF model is part of the commercially available TREAT Lab software, available from Treat Systems.

## References

[1] Henriksen DP, Pottegård A, Laursen CB, Jensen TG, Hallas J, Pedersen C (2015). Risk factors for hospitalization due to community-acquired sepsis—a population-based case-control study. PLoS ONE.

[2] Timbrook TT, Spivak ES, Hanson KE (2018). Current and future opportunities for rapid diagnostics in antimicrobial stewardship. Med Clin N Am.

[3] Florio W, Morici P, Ghelardi E, Barnini S, Lupetti A (2018). Recent advances in the microbiological diagnosis of bloodstream infections. Crit Rev Microbiol.

[4] Peker N, Couto N, Sinha B, Rossen JW (2018). Diagnosis of bloodstream infections from positive blood cultures and directly from blood samples: recent developments in molecular approaches. Clin Microbiol Infect.

[5] Mangioni D, Viaggi B, Giani T, Arena F, D’Arienzo S, Forni S (2019). Diagnostic stewardship for sepsis: the need for risk stratification to triage patients for fast microbiology workflows. Future Microbiol.

[6] Bates DW (1991). Contaminant blood cultures and resource utilization. JAMA.

[7] Coburn B, Morris AM, Tomlinson G, Detsky AS (2012). Does this adult patient with suspected bacteremia require blood cultures?. JAMA.

[8] Eliakim-Raz N, Bates DW, Leibovici L (2015). Predicting bacteraemia in validated models—a systematic review. Clin Microbiol Infect.

[9] Ward L, Andreassen S, Astrup JJ, Rahmani Z, Fantini M, Sambri V (2019). Clinical- vs. model-based selection of patients suspected of sepsis for direct-from-blood rapid diagnostics in the emergency department: a retrospective study. Eur J Clin Microbiol Infect Dis.

[10] Royal College of Physicians (2017). National Early Warning Score (NEWS) 2: standardising the assessment of acute-illness severity in the NHS.

[11] Shapiro NI, Wolfe RE, Moore RB, Smith E, Burdick E, Bates DW (2003). Mortality in Emergency Department Sepsis (MEDS) score: a prospectively derived and validated clinical prediction rule. Crit Care Med.

[12] Bone RC, Balk RA, Cerra FB, Dellinger RP, Fein AM, Knaus WA (1992). Definitions for sepsis and organ failure and guidelines for the use of innovative therapies in sepsis. The ACCP/SCCM Consensus Conference Committee. American College of Chest Physicians/Society of Critical Care Medicine. CHEST J.

[13] Singer M, Deutschman CS, Seymour CW, Shankar-Hari M, Annane D, Bauer M (2016). The third international consensus definitions for sepsis and septic shock (Sepsis-3). JAMA.

[14] Shapiro NI, Wolfe RE, Wright SB, Moore R, Bates DW (2008). Who needs a blood culture? A prospectively derived and validated prediction rule. J Emerg Med.

[15] Ward L, Møller JK, Eliakim-Raz N, Andreassen S (2018). Prediction of bacteraemia and of 30-day mortality among patients with suspected infection using a CPN model of systemic inflammation. IFAC-PapersOnLine.

[16] Poses RM, Anthony M (1991). Availability, wishful thinking, and physicians’ diagnostic judgments for patients with suspected bacteremia. Med Decis Mak.

[17] Pawlowicz A, Holland C, Zou B, Payton T, Tyndall JA, Allen B (2016). Implementation of an evidence-based algorithm reduces blood culture overuse in an adult emergency department. Gen Intern Med Clin Innov.

[18] Jessen MK, Mackenhauer J, Hvass AMSW, Ellermann-Eriksen S, Skibsted S, Kirkegaard H (2016). Prediction of bacteremia in the emergency department. Eur J Emerg Med.

[19] Arboe B, Laub RR, Kronborg G, Knudsen JD (2014). Evaluation of the decision support system for antimicrobial treatment, TREAT, in an acute medical ward of a university hospital. Int J Infect Dis.

[20] Ward LM, Møller J, Østergaard C, Mogensen M, Paul M, Leibovici L, et al. Prediction of bacteraemia in a low-bacteraemia-prevalence cohort using the Treat decision support system. In: Conference of The International Society for Medical Innovation and Technology, iSMIT. Baden-Baden. 2013.

[21] Paul M, Andreassen S, Tacconelli E, Nielsen AD, Almanasreh N, Frank U (2006). Improving empirical antibiotic treatment using TREAT, a computerized decision support system: cluster randomized trial. J Antimicrob Chemother.

[22] Ward L, Paul M, Andreassen S (2017). Automatic learning of mortality in a CPN model of the systemic inflammatory response syndrome. Math Biosci.

[23] DeLong ER, DeLong DM, Clarke-Pearson DL (1988). Comparing the areas under two or more correlated receiver operating characteristic curves: a nonparametric approach. Biometrics.

[24] Hunter JD (2007). Matplotlib: a 2D graphics environment. Comput Sci Eng.

[25] Perl B, Gottehrer NP, Raveh D, Schlesinger Y, Rudensky B, Yinnon AM (1999). Cost-effectiveness of blood cultures for adult patients with cellulitis. Clin Infect Dis.

[26] National Institute for Health and Care Excellence. Tests for rapidly identifying bloodstream bacteria and fungi (LightCycler SeptiFast Test MGRADE, SepsiTest and IRIDICA BAC BSI assay). 2016. https://www.nice.org.uk/guidance/dg20/chapter/4-Outcomes. Accessed 28 2017.

[27] Seymour CW, Liu VX, Iwashyna TJ, Brunkhorst FM, Rea TD, Scherag A (2016). Assessment of clinical criteria for sepsis. JAMA.

[28] Zhao J, He Y, Xu P, Liu J, Ye S, Cao Y (2020). Serum ammonia levels on admission for predicting sepsis patient mortality at D28 in the emergency department. Medicine (Baltimore).

[29] Xia Y, Zou L, Li D, Qin Q, Hu H, Zhou Y (2020). The ability of an improved qSOFA score to predict acute sepsis severity and prognosis among adult patients. Medicine (Baltimore).

[30] Gonzalez Del Castillo J, Wilson DC, Clemente-Callejo C, Román F, Bardés-Robles I, Jiménez I (2019). Biomarkers and clinical scores to identify patient populations at risk of delayed antibiotic administration or intensive care admission. Crit Care.

[31] Kim H, Hur M, Struck J, Bergmann A, Di Somma S (2019). Circulating biologically active adrenomedullin predicts organ failure and mortality in sepsis. Ann Lab Med.

[32] Saeed K, Wilson DC, Bloos F, Schuetz P, van der Does Y, Melander O (2019). The early identification of disease progression in patients with suspected infection presenting to the emergency department: a multi-centre derivation and validation study. Crit Care.

[33] Abdullah SMOB, Sørensen RH, Dessau RBC, Sattar SMRU, Wiese L, Nielsen FE (2019). Prognostic accuracy of qSOFA in predicting 28-day mortality among infected patients in an emergency department: a prospective validation study. Emerg Med J.

[34] Yu H, Nie L, Liu A, Wu K, Hsein YC, Yen DW (2019). Combining procalcitonin with the qSOFA and sepsis mortality prediction. Medicine (United States).

[35] Prabhakar SM, Tagami T, Liu N, Samsudin MI, Ng JCJ, Koh ZX (2019). Combining quick sequential organ failure assessment score with heart rate variability may improve predictive ability for mortality in septic patients at the emergency department. PLoS ONE.

[36] García-Lamberechts EJ, Martín-Sánchez FJ, Julián-Jiménez A, Llopis F, Martínez-Ortizde Zarate M, Arranz-Nieto MJ (2018). Infection and systemic inflammatory response syndrome in older patients in the emergency department: a 30-day risk model. Emergencias Rev la Soc Esp Med Emergencias.

[37] Li D, Zhou Y, Yu J, Yu H, Xia Y, Zhang L (2018). Evaluation of a novel prognostic score based on thrombosis and inflammation in patients with sepsis: a retrospective cohort study. Clin Chem Lab Med.

[38] Zhao Y, Jia Y, Li C, Fang Y, Shao R (2018). The risk stratification and prognostic evaluation of soluble programmed death-1 on patients with sepsis in emergency department. Am J Emerg Med.

[39] Innocenti F, Tozzi C, Donnini C, De Villa E, Conti A, Zanobetti M (2017). SOFA score in septic patients: incremental prognostic value over age, comorbidities, and parameters of sepsis severity. Intern Emerg Med.

[40] Wang J-Y, Chen Y-X, Guo S-B, Mei X, Yang P (2016). Predictive performance of quick Sepsis-related Organ Failure Assessment for mortality and ICU admission in patients with infection at the ED. Am J Emerg Med.

[41] Mirijello A, Tosoni A, Zaccone V, Impagnatiello M, Passaro G, Vallone CV (2019). MEDS score and Vitamin D status are independent predictors of mortality in a cohort of Internal Medicine patients with microbiological identified Sepsis. Eur Rev Med Pharmacol Sci.

[42] Brink A, Alsma J, Verdonschot RJCG, Rood PPM, Zietse R, Lingsma HF (2019). Predicting mortality in patients with suspected sepsis at the Emergency Department; A retrospective cohort study comparing qSOFA, SIRS and National Early Warning Score. PLoS ONE.

[43] Ramos JGR, da Hora Passos R, Teixeira MB, Gobatto ALN, Coutinho RVdS, Caldas JR (2018). Prognostic ability of quick-SOFA across different age groups of patients with suspected infection outside the intensive care unit: a cohort study. J Crit Care.

